# Preclinical exploration and current clinical applications of immunotherapeutic strategies for hepatocellular carcinoma

**DOI:** 10.3389/fimmu.2026.1769251

**Published:** 2026-02-10

**Authors:** Haoyang Yu, Xin Wen, Mengying Cui, Shui Liu

**Affiliations:** 1 Department of Hepatobiliary and Pancreatic Surgery, The Second Hospital of Jilin University, Changchun, China; 2 Department of Clinical Laboratory, The Second Hospital of Jilin University, Jilin University, Changchun, China

**Keywords:** antitumor mechanisms, clinical applications, hepatocellular carcinoma, immunotherapy, preclinical exploration

## Abstract

Hepatocellular carcinoma (HCC) is the sixth most common cancer and the third leading cause of cancer death worldwide. Treatment of HCC has shifted from traditional modalities to immunotherapy-centered combination strategies. While the current immunotherapies can substantially reduce the risk of death for patients with HCC, overall survival improvement is limited because of tumor heterogeneity and an immunosuppressive microenvironment. Moreover, the widespread application of these treatments is challenged by high costs, drug resistance, and frequent adverse events. This review outlines the mechanisms of the available HCC immunotherapeutics and summarizes the preclinical explorations of these treatments. We also describe the current clinical applications and underlying mechanisms and discuss issues of resistance and heterogeneity. We further provide an overview of emerging approaches against cancer platforms in HCC, aiming to provide practical references for clinical immunotherapy of HCC.

## 1 Introduction

Primary liver cancer is one of common digestive system malignancies, and ranks sixth in incidence and third in mortality among all cancers In the WHO 2022 statistics (1). Hepatocellular carcinoma (HCC) accounting for approximately 75%-85% of all primary liver cancers (2, 3). Currently, curative surgical resection is the only potentially curative option besides liver transplantation for HCC. In recent years, the 5-year survival rate for HCC has improved from less than 18% to 22%, as a result of the development of multimodal treatment frameworks incorporating ablation, transarterial therapies, and tyrosine kinase inhibitors (TKIs) (4). However, outcomes remain concerning for patients with unresectable disease and those at high risk of recurrence. Approximately 70% of HCC patients are first diagnosed at an intermediate or advanced stage, and 50~70% of HCC patients who undergo surgery face a risk of recurrence, which severely compromises prognosis (5).

Since the approval of the immune checkpoint inhibitor (ICI) nivolumab for intermediate-to-advanced HCC (6), immunotherapy has produced breakthrough advances (7). Immunotherapy reduces the risk of death by approximately 22%–43% and extends survival by more than six months on average in HCC (8). However, significant challenges remain. Tumor cells can develop adaptive resistance through epigenetic and metabolic reprogramming (9), and amplification of the adenosine axis and VEGF-mediated immunosuppression within the tumor microenvironment (TME) fosters immune tolerance (10). Moreover, the lack of effective biomarkers hampers drug selection for patients of HCC, and the widely used regimen atezolizumab plus bevacizumab can cause gastrointestinal bleeding, portal hypertension, and hepatic decompensation with appreciable frequency (11). The high cost and less than optimal effectiveness of immunotherapy also limits its application (12).

**Figure 1:**
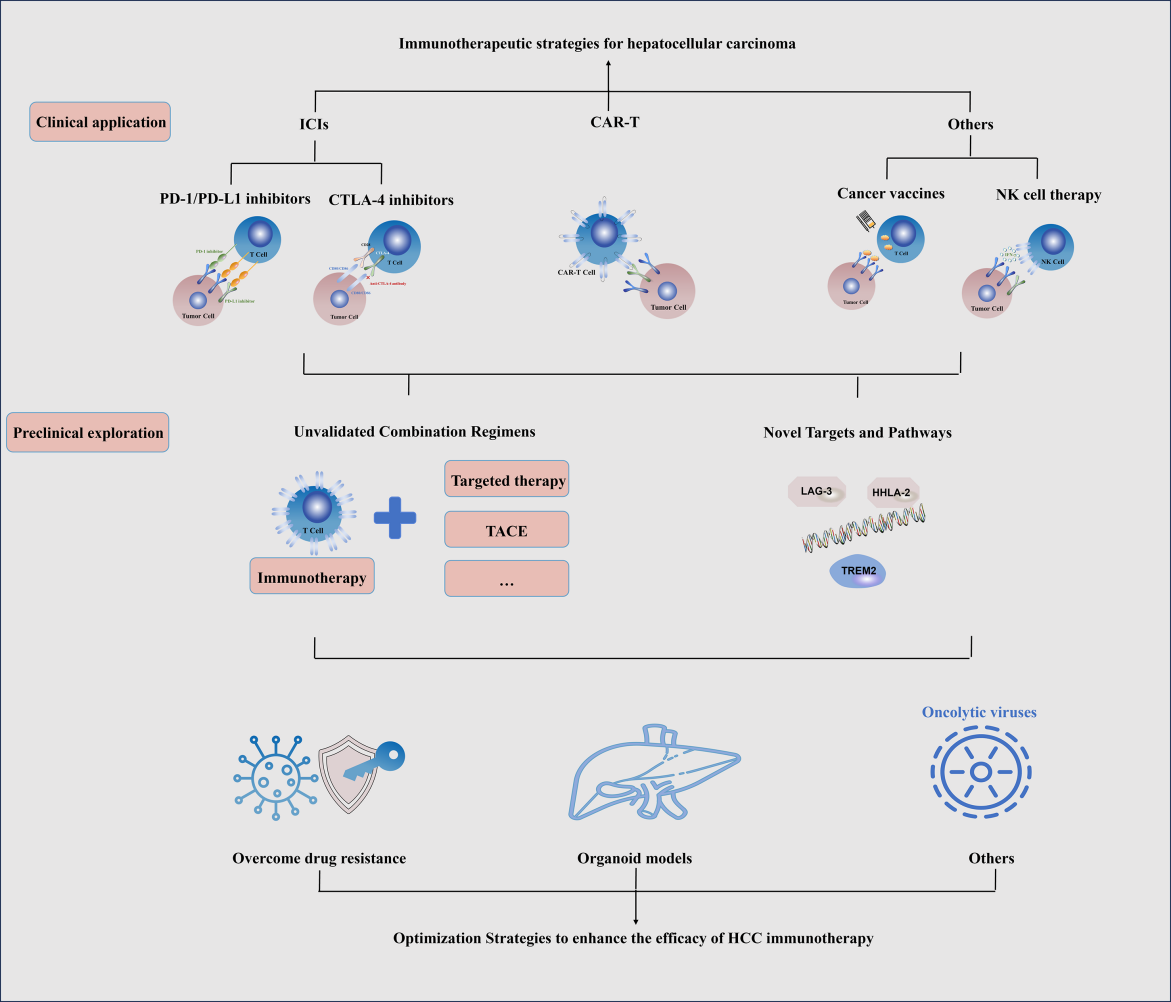
Schematic diagram of clinical application and preclinical exploration of immunotherapy for HCC.

In the manuscript, we comprehensively describe the mechanisms and current clinical application of immunotherapy for HCC and summarize the preclinical advances, challenges, and potential strategies, with the aim to provide a systematic reference for basic research and clinical practice in this field, facilitate the development and optimization of new immunotherapy regimens, and offer ideas for addressing bottleneck issues in clinical application (such as drug resistance, insufficient efficacy prediction), ultimately improving the prognosis and quality of life of patients with HCC (**Figure 1**).

## 2 The antitumor mechanisms of immunotherapy in HCC

### 2.1 Immune checkpoint inhibitors

**Figure 2:**
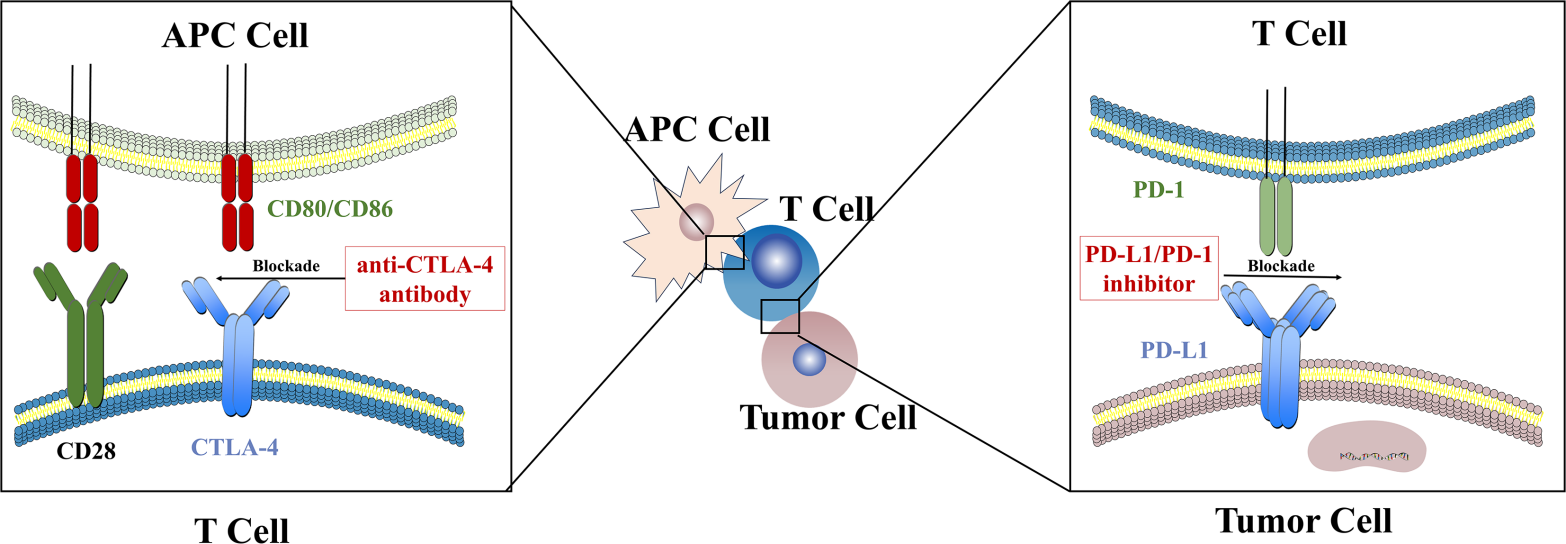
The antitumor mechanisms of immune checkpoint inhibitors in HCC.

Immune checkpoint molecules are pivotal regulators of immune homeostasis and T-cell activity. However, in the tumor TME, the overexpression of inhibitory checkpoints constitutes a primary mechanism of immune evasion (13). This overexpression frequently leads to T-cell exhaustion, thereby facilitating tumor growth. Consequently, immune checkpoint inhibitors have emerged as a critical therapeutic strategy in current cancer treatment (14). The use of immune checkpoint inhibitors (ICIs), such as inhibitors against programmed cell death protein 1(PD-1)/programmed cell death ligand 1 (PD-L1) or cytotoxic T-lymphocyte antigen 4 (CTLA-4), to enhance costimulation and relieve immunosuppression has become a first-line approach for the treatment of many cancers (15). Most HCC cases are accompanied by fibrosis or cirrhosis because of chronic necro-inflammation (10). This pathological background significantly influences immunotherapeutic efficacy. The fibrotic microenvironment acts as a physical barrier that restricts T-cell infiltration, while activated hepatic stellate cells secrete transforming growth factor-beta (TGF-B) to foster an immunosuppressive milieu, thereby limiting the antitumor activity of immune checkpoint inhibitors (16). Moreover, because of the liver’s unique immune microenvironment, the mechanisms of immunotherapy in HCC are particularly complex and distinctive (17) (**Figure 2**). In HCC tumors, PD-1 is highly expressed on exhausted intratumoral CD8^+^ T cells; blocking PD-1 interrupts SHP-2 recruitment to phosphorylated ITIM/ITSM motifs, thereby restoring proximal CD3ζ/ZAP70 and CD28 signaling, reinvigorating effector cytokine production and cytotoxicity in HCC T cells, and increasing secretion and effector functions of IL-2 and other cytokines (18). PD-1/PD-L1 blockade in HCC also preserves and expands a TCF1^+^ progenitor-exhausted CD8^+^ subset linked to better response potential; this reinvigoration is further enhanced when additional inhibitory axes (e.g., TIGIT) are relieved in ex vivo HCC samples (19). Anti-PD-L1 antibodies exert similar effects. Additionally, the Fc region of some anti-PD-L1 antibodies engages Fcγ receptors to activate natural killer (NK) cells and trigger antibody-dependent cellular cytotoxicity, directly eliminating PD-L1-high tumor cells or suppressive myeloid cells (20). Those anti-PD-L1 antibodies also influence tumor AKT/mTOR signaling, glycolysis, apoptosis, and autophagy and can modulate gene transcription (21).

In the HCC TME, enrichment of tumor-infiltrating regulatory T cells (Tregs) together with the upregulation of CTLA-4 collectively attenuate costimulatory signaling. CTLA-4, another receptor on T cells, competes with CD28 for CD80/CD86 on antigen-presenting cells (APCs) and thereby dampens immunostimulatory signaling (22). Anti-CTLA-4 agents block this process and partially restore co-stimulation. Mechanistically, B7 molecules on activated T cells are cleared from the membrane by CTLA-4 in the tumor milieu in HCC (23). Inhibiting CTLA-4 increases both the quantity and quality of activated T cells. Because much of the efficacy of anti-CTLA-4 approaches is derived from Fc-mediated depletion of Tregs (24), molecularly enhancing Fc functions can markedly augment Treg clearance and antitumor responses. LAG-3, another checkpoint molecule expressed on tumor-infiltrating T cells, uses a distinctive geometric configuration to enforce tight membrane spacing between T cells and APCs, thereby impeding immune activation (25). Anti-LAG-3 antibodies disrupt this association, restore TCR/CD4 signaling, and reinstate antigen presentation (26).

### 2.2 Anti-angiogenic agents

HCC is a highly vascularized tumor. Multiple axes, including VEGF/VEGFR, FGF, PDGF, and Ang/Tie2 signaling, drive endothelial hyperproliferation with poor pericyte coverage, producing an aberrant, leaky, tortuous microvasculature (27). This creates local hypoxia that disables oxygen-dependent therapies; the distorted capillary architecture elevates interstitial pressure and leakage, further impeding drug penetration into the tumor core (28). Anti-VEGF antibodies increase pericyte coverage, reduce permeability, and raise oxygenation (29), thereby improving luminal patency and effective perfusion, lowering interstitial pressure, and increasing the intratumoral concentrations and efficacy of cytotoxics (30). STING agonists also repair abnormal vasculature: by inducing robust type-I interferon and chemokine release, they remodel the TME through mechanisms akin to anti-VEGF therapy, promoting vascular normalization (31). The ability of these agonists to activate APCs and dendritic cells has likewise made them a research focus (32).

### 2.3 Other immunotherapeutic strategies

Several other anti-tumor mechanisms have gained attention as potential therapeutic targets in recent years. B cells are key constituents of the TME and closely linked to tertiary lymphoid structures (TLS) within tumors (33). Clonal expansion and affinity maturation of B cells within TLS synergize with enhanced CD8^+^ T-cell effector functions; TLS-rich tumors tend to mount more active immune responses (34). The combination of FOLFOX-HAIC with immune checkpoint blockade can remodel the TME and promote TLS formation (35). However, the relationship between dynamic TLS changes and treatment outcomes in HCC requires confirmation in larger cohorts or experimental studies.

Hepatic Kupffer cells, sinusoidal endothelial cells, and dendritic cells are sensitive to metabolites from the gut microbiota, especially secondary bile acids, which can positively influence responses to immune checkpoint inhibitors (36). Probiotics and healthy microbiota enhance APC function, upregulate CXCL9/10 and adhesion molecules, and facilitate CD8^+^ T-cell infiltration into tumors (37). These results suggest that dietary modulation may confer therapeutic benefits in HCC.

## 3 Current immunotherapy for HCC in clinical

### 3.1 Immune checkpoint inhibitors

PD1 inhibitors and PD-L1 inhibitors, as the core of currently recommended treatment regimens for HCC, are mostly used in combination therapies. The combination of atezolizumab (a PD-L1 inhibitor) and bevacizumab (an anti-VEGF agent) has become a first-line treatment for unresectable advanced HCC (38). Their complementary functions in relieving immunosuppression enable them to achieve a synergistic effect in practical application, establishing this combination as the first-line treatment standard for advanced unresectable HCC (39).

The combination of PD1 inhibitors with anti-angiogenic agents also yields positive effects. The combination of camrelizumab and rivoceranib synergistically enhances immune function and repairs the microenvironment (40, 41). An additional advantage of this treatment is that vascular normalization promotes the formation of TLS, allowing continuous infiltration of peripheral T cells into tumors, which consolidates treatment efficacy and improves prognosis (42). Some multi-target TKIs have unique effects. Lenvatinib downregulates the Treg/TAM pathway and enhances the IFN-I antigen presentation pathway (43). However, its clinical application scope is narrower than that of bevacizumab, so it is recommended as an alternative treatment. Cabozantinib is similar in this regard: in addition to its anti-angiogenic role, it also interferes with the immunosuppressive loop of MET/AXL-myeloid-epithelial-mesenchymal transition (44). However, phase III clinical results for cabozantinib in HCC show unstable survival benefits (45). Notably, not all combinations of PD1/PD-L1 inhibitors with anti-angiogenic agents achieve favorable outcomes; excessive vascular reduction may occur when some highly potent TKIs are used or when the treatment sequence is incorrect (46), offsetting the synergistic effect with immune checkpoint inhibitors. Therefore, in clinical drug selection, in addition to efficacy, factors such as the patient’s genotype, biological matching degree, treatment timing and dosage, and the impact of subsequent treatment on the current regimen should be considered (47).

CTLA-4 increases the number of naïve lymphocytes, so it can complement immune checkpoint inhibitors (8). Clinically, a high single dose of tremelimumab combined with regular durvalumab is commonly used. This treatment does not directly affect blood vessels, and thus it is suitable for patients at high risk of bleeding or those ineligible for anti-VEGF therapy (48). It is also one of the most commonly used combinations in clinical practice.

The combination of immune checkpoint inhibitors and other treatment regimens, such as transarterial chemoembolization (TACE), is also widely used in clinical practice (49). TACE enhances antigen presentation and alleviates hypoxia by improving perfusion, facilitating T-cell infiltration (50). Durvalumab combined with TACE has significant advantages over ablation alone or immune checkpoint inhibitors alone; anti-VEGF agents can also be added to enhance the synergistic effect when necessary (51). This treatment regimen is often used in cases where local treatment is possible but the tumor burden is large (52). Another retrospective analysis by Rui Zhu et al. showed that Combination therapy regimen of rivoceranib-camrelizumab with TACE achieved significantly higher partial (39.8% vs. 20.2%, P = 0.006) and objective response rates (50.6% vs. 29.7%, P = 0.006) than systemic therapy alone (53). Similar to other combination therapies, attention should be paid to the rationality of treatment timing and subsequent treatment.

### 3.2 Chimeric antigen receptor T-cell immunotherapy

Chimeric antigen receptor T(CAR-T) - cell immunotherapy, which reprograms T cells to recognize antigens independently of MHC, has revolutionized the treatment of hematologic malignancies. Although its application in solid tumors is challenged by the immunosuppressive TME and physical barriers, CAR-T therapy targeting liver-specific antigens has emerged as a promising strategy for HCC (54). Since the expression of glypican-3 (GPC3) in HCC cells is significantly higher than that in normal liver tissue (55). GPC3-targeting CAR-T cell therapy has high specificity and good safety, and it is currently used in some specific clinical scenarios. In clinical practice, CAR-T cells are usually modified for tumor treatment. CAR-T cells engineered to overexpress the transcription factor RUNX3 (which is associated with immune memory) prolong the retention these CAR-T cells (specifically CT017) in tumor tissue without significant safety differences (56). Conjugating alpha-fetoprotein (AFP, an intracellular antigen) to CAR-T cells to form a dual-antigen-targeting CAR-T cell therapy significantly expanded the killing range of the drug, extending the recognized antigens from the cell surface to the intracellular space (57). However, this approach entails inherent safety risks, particularly the potential for ‘on-target, off-tumor’ toxicity in regenerating hepatocytes that transiently express AFP, or cross-reactivity with similar peptides presented on the surface of vital organs. Therefore, more specific antigens need to be identified in clinical practice to avoid damage to normal tissue. Additionally, the modification of CAR-T cells with CD133 not only enhances tumor killing but also affects angiogenesis, influencing tumor blood supply and invasion (58), which can fundamentally reverse HCC drug resistance. However, clinical application of this therapy is challenging because of its low specificity, as CD133 is also expressed in normal hepatocytes.

Combining CAR-T cell therapy with other treatment regimens is also an option in clinical treatment. TACE can induce tumor cells to release a large number of surface antigens, and the increased antigen supply provides sufficient signals for CAR-T cell therapy (59), enhancing its targeting effect. Immune checkpoint inhibitors can also be added to assist treatment when necessary. While the application of CAR-T cell therapy and inhibitors in HCC is more difficult than in other tumors because of the unique TME (60), satisfactory clinical effects can still be achieved through modifying the drugs themselves or combining them with other regimens. However, these treatment regimens require careful design and strict evaluation for clinical application.

### 3.3 Other immunotherapeutic strategies

In addition to the widely used regimens mentioned above, several cutting-edge immunotherapies have also shown clinical potential. Cancer vaccines have been engineered to deliver specific antigens expressed on HCC cells via platforms such as DNA or peptides (61). These antigen vaccines have strong adaptability and can be combined with various other treatment regimens. For example, the combination of an antigen vaccine with interleukin-12 (IL-12) plasmid and pembrolizumab achieved an objective response rate of 30%, significantly enhancing tumor-specific immune capacity and improving clinical response (62). During local treatment, antigen vaccines also enhance cross-presentation and antigen-specific responses (such as responses to AFP), thereby improving treatment benefits (63).

NK cell therapy, which involves the infusion of autologous or allogeneic NK cells or cytokine-sensitized NK cells, also has clinical activity and high safety (64). This regimen is particularly effective for patients with T-cell exhaustion or strong immunosuppression. For patients with poor liver function or advanced HCC during the peri-interventional period, the infusion of invariant NK T cells can result in rapid secretion of interferon-gamma (IFN-γ), activation of dendritic cells and NK cells, reshaping of myeloid suppression, and significantly improvement in patients’ quality of life (65). The combination of oncolytic viruses injected into tumor tissue or via the hepatic artery with TACE can result in tumor cell lysis while enhancing immunity; this is also an innovative clinical treatment method used during the peri-interventional period (66).

## 4 Preclinical exploration

### 4.1 Unvalidated combination therapy regimens

While clinical applications have established standard regimens, preclinical research and early-phase trials focus on addressing unmet needs such as drug resistance and limited applicability (**Table 1**). In addition to the first-line therapy of atezolizumab plus bevacizumab, which can significantly improve OS compared with sorafenib (39), the combination of camrelizumab and rivoceranib also significantly prolongs patient survival (67). Supported by the phase III Cares-310 trial, this combination achieves a median OS of 22.1 months versus 15.2 months for sorafenib, with vascular normalization as a key mechanism enhancing T-cell infiltration (67).It is preferred for patients with intermediate liver function (Child-Pugh A/B7), especially those with vascular invasion but low bleeding risk (e.g., no active varices). For NASH-related HCC, which is often associated with metabolic disorders and a “cold” TME, the anti-angiogenic effect of rivoceranib may better remodel the immunosuppressive microenvironment compared to other TKIs (45). The 4-year OS update from the HIMALAYA trial confirms durable survival benefit, with a 31% 4-year OS rate in the tremelimumab (single high dose) + durvalumab group (68). This regimen is the first choice for patients at high risk of bleeding (e.g., active varices, recent gastrointestinal bleeding) or those ineligible for anti-VEGF therapy. It also shows promising efficacy in HCV-related HCC, possibly due to CTLA-4 blockade-induced depletion of Tregs in virus-associated inflammatory microenvironments (10).

**Table 1:** The current advances of ICIs for HCC based on clinical trials.

Experiment treatment	Control treatment	Number of patients	Efficacy	References
Atezolizumab plus Bevacizumab	Sorafenib	336 vs. 165	OS (at 12 months): 67.2% vs. 64.0% mPFS: 6.8 vs. 4.3 months	(39)
Single Tremelimumab Regular Interval Durvalumab	Durvalumab vs. Sorafenib	453 vs. 453 vs. 445	mOS: 17.35 vs. 16.76 vs.13.63 months mPFS: 3.78 vs. 3.58 vs. 3.91 months OS (at 12 months): 60.3% vs. 60.1% vs. 55.2% OS (at 24 months): 41.4% vs. 39.0% vs. 33.0 OS (at 36 months): 31.0% vs. 25.7% vs. 21.0%	(115)
Nivolumab plus ipilimumab	Lenvatinib/Sorafenib	335 vs. 333	mOS: 23.7 vs. 20.6 months OS (at 24 months): 49.0% vs. 39.0% OS (at 36 months): 38.0% vs. 24.0%	(116)
Camrelizumab plus Rivoceranib	Sorafenib	272 vs. 271	mOS: 22.1 vs. 15.2 months mPFS: 5.6 vs. 3.7 months	(67)
Sintilimab plus IBI305	Sorafenib	380 vs. 191	OS: 10.4 vs 8.5 months mPFS: 4.6 vs 2.8 months	(117)
Nivolumab	Sorafenib	371 vs. 372	mOS: 16.4 vs 14.7 months mPFS: 7.5 vs 5.7 months	(118)
Pembrolizumab monotherapy	Placebo	278 vs. 135	mOS: 13.9 vs 10.6 months mPFS: 3.0 vs 2.8 months	(119)
Camrelizumab plus Apatinib	/	190	OS (at 12 months): 74.7% (First line setting) vs. 68.2% (Second line setting) mPFS: 5.7 months (First line setting) vs. 5.5 months (Second line setting)	(74)
Cabozantinib plus atezolizumab	Sorafenib	432 vs.217	mPFS: 6.8 vs. 4.2 months mOS: 15.4 vs. 15.5 months	(70)
iNKT Cells plus Embolization	TACE	27 vs. 27	mPFS: 5.7 vs. 2.7 months	(65)
Toripalimab plus bevacizumab	Sorafenib	162 vs. 164	mPFS: 5.8 vs. 4.0 months mOS: 20.0 vs. 14.5 months	(120)
Anlotinib plus penpulimab	Sorafenib	433 vs. 216	mPFS: 6.9 vs. 2.8 months mOS: 16.5 vs. 13.2 months	(121)
Pembrolizumab plus lenvatinib	Lenvatinib plus placebo	395 vs. 399	mPFS: 8.2 vs. 8.0 months mOS: 21.2 vs. 19.0 months	(69)

However, not all evaluated drugs have achieved the therapeutic goals. For example, the combination of lenvatinib and pembrolizumab did not significantly improve OS or progression-free survival (PFS) (69). While the combination of cabozantinib and atezolizumab resulted in improved PFS, it did not improve OS (70). Even when combined with local ablation therapy, the combination of durvalumab and bevacizumab only increased PFS compared with TACE alone, but whether OS changes remain under follow-up observation (71). Additionally, some regimens have not yet passed full phase III clinical trials. While they cannot be temporarily used in actual clinical treatment, they can provide new ideas for researchers. Yau et al. discovered the therapeutic potential of the nivolumab plus cabozantinib combination through early exploration (72). The researchers also found that the combination of nivolumab and ipilimumab alleviates symptoms in patients who have received previous treatment (73). Xu et al. confirmed the favorable safety and efficacy of the camrelizumab plus apatinib combination in a phase II single-arm clinical trial (74).

Despite the increase in research on new combination therapy strategies, some of these treatments are associated with safety issues (75). Therefore, some drugs not yet recommended by HCC treatment guidelines also deserve attention, as they have the potential to be combined with immune checkpoint inhibitors. Histological evidence has shown that high expression of LAG-3 is associated with poor prognosis in HCC (76). Guo et al. found that inhibiting the fibrinogen-like protein 1 (FGL1)-LAG-3 axis produces complementary effects with PD-1/PD-L1 inhibitors (77), and this strategy has therapeutic potential for HCC. Sun et al. found that the platelet-mediated CD155-TIGIT signaling enables circulating tumor cells to escape the killing effect of NK cells, which indicates that TIGIT blockade can inhibit the immune evasion of tumor cells (78). Hsiehchen et al. further found that the combination of TIGHT pathway-related interventions with PD-1 inhibitors alleviates the dual-pathway inhibition of T cells and NK cells (79). This regimen has demonstrated clinical feasibility and promising antitumor activity, thus having the potential to solve the problem of immune resistance in HCC.

### 4.2 Novel targets and pathways for immunotherapy

In addition to these attempts to expand treatments based on existing combination regimens, some research teams are conducting clinical trials on treatments based on other mechanisms of action. Extending the findings that C-X-C chemokine receptor type 2 (CXCR2) alleviates immunosuppression when combined with immune checkpoint inhibitors through the neutrophil/macrophage axis, Myojin et al. speculated that the combination of inhibitors against adenosine A2A receptor (A2A) and anti-PD-1 agents might produce a strong synergistic effect in suppressing tumor growth and prolonging survival (80), which might target NASH-related HCC patients with high adenosine levels (indicated by elevated plasma adenosine concentration), who often respond poorly to conventional ICI therapy. This combination also has an independent antitumor function in non-alcoholic steatohepatitis-related HCC.

The discovery that ferroptosis in tumor cells can alleviate immunosuppression has also attracted attention. Cheu et al. proposed the following strategy: triggering ferroptosis through inhibitors targeting ferroptosis suppressor protein 1 (FSP1), upregulating antigen presentation by dendritic cells/macrophages and T-cell effector function, and combining this approach with PD-1/PD-L1 inhibitors or CD40 agonists to significantly prolong survival (81). Meng et al. triggered ferroptosis by injecting a composite hydrogel of imipenem and sulfasalazine; when combined with anti-PD-1 agents, this treatment significantly alleviated symptoms and induced immune memory in murine HCC models with malignant ascites (82). Wu et al. found that gut microbiome biomarkers are associated with the response of patients with HCC to anti-PD-1-based immunotherapy (83). This discovery provides a direct link between the gut microbiome and immunotherapy outcome, offering new possibilities for non-invasive biomarker candidates to predict therapeutic efficacy.

Several studies on other targets and pathways have yielded promising possibilities. A recent study by the team led by Huang et al. identified that HHLA2 activates the c-Met signaling pathway, thereby serving as a potential therapeutic target and providing a rationale for designing new combination therapies (84). Through studying the important role of triggering receptor expressed on myeloid cells 2 (TREM) in the immunosuppressive mechanism of nonalcoholic steatohepatitis-related HCC, Wang et al. confirmed that targeting TREM2 can alleviate immunosuppression in steatohepatitis and exert a synergistic effect with T-cell checkpoint inhibitors (85). Targets such as LAG-3, T-cell immunoglobulin and mucin domain-containing protein 3 (TIM-3), sialic acid-binding immunoglobulin-like lectin 15 (Siglec-15), CD24/Siglec-10, and CD47/signal regulatory protein alpha (SIRPα) can all alleviate immunosuppression in HCC to a certain extent t (86–88).

## 5 Dilemmas and prospects

### 5.1 Drug resistance of existing immunotherapies

The expansion of immune checkpoint inhibitors, as the main approach for HCC immunotherapy, and the development of combination treatment regimens have been crucial for the improvements in therapeutic efficacy and outcome for patients with HCC (89). However, there are still dilemmas in clinical application. Drug resistance of HCC to immunotherapy has long been the most critical factor leading to poor clinical efficacy and is classified into primary resistance and acquired resistance. The WNT/B-catenin pathway is a core signaling pathway for human embryonic development and tissue repair (90). Under normal conditions, it regulates cell proliferation and differentiation; however, this pathway is continuously activated in tumor cells and is a mechanism of primary drug resistance (91). This type of resistance alters the TME, making the efficacy of many HCC immunotherapy combination regimens, including atezolizumab plus bevacizumab, far less effective than in other tumors (92). To address this dilemma, the combination of LNP-siRNA targeting B-catenin and immune checkpoint inhibitors can inhibit CTNNB1 to a certain extent, but this approach has issues such as insufficient specificity, making it difficult to apply in actual clinical scenarios (93).

Another mechanism of primary resistance lies in immune exhaustion caused by hypoxia-vascular abnormalities in the TME (94). Hypoxia upregulates PD-L1 via HIF-1α to promote immune escape. Therefore, it is necessary to correct the hypoxia issue during treatment. Vascular changes also play a similar role: VEGF induces dendritic cell maturation disorders and Treg expansion and reduces the responsiveness of endothelial cells (95). This is also a strategy in existing treatment regimens. Overall, the complexity of primary resistance means that treatment strategies need to take many factors into account, often failing to achieve the desired effect.

Acquired resistance is another critical issue that requires attention. The myeloid-dominated immunosuppressive microenvironment is not only associated with primary resistance but may also be associated with acquired resistance. In HCC, SPP1^+^ macrophages and cancer-associated fibroblasts form an immune barrier, which traps CD8^+^ T cells outside the tumor nest and significantly reduces the immune response (96). Inhibiting TREM2 enhances the efficacy of anti-PD-L1, indicating that TREM2 is a key target in the inhibitory mechanism (97). The inhibition of the SPP1^+^ macrophage-stem cell-like tumor axis is currently an approach to address the drug-resistant TME. Intervening in SPP1^+^ macrophages and TREM2^+^ macrophages through pharmacological or genetic means has shown good effects in various tumor SPP1^+^ (98), but its efficacy in HCC still requires further research.

The strong heterogeneity of HCC is another reason for the difficulty in formulating treatment regimens. The copy number variation in HCC conforms to a two-stage model of “interspersed bursts + gradual accumulation”, in which a prolonged phase of gradual accumulation leads to higher tumor heterogeneity and rapid recurrence (99). Early key driver mutations (such as TP53 and CTNNB1 mutations) lay the foundation for different branches of tumor evolution, while late-stage copy number variation and viral integration shape different transcriptional subtypes (100). This explains why it is particularly difficult to formulate reasonable treatment strategies for HCC, especially in the advanced stage.

### 5.2 Organoid models

To improve the targeting of treatment regimens and reduce drug resistance, the use of cells derived from patient tumor tissues to establish tumor organoids for research has become a hotspot in recent years. This approach facilitates genetic-level intervention in tumor treatment and significantly improves drug efficacy (101). Organoid therapy for brain tissue diseases has achieved wide clinical application (102). A similar biobank has also been established for HCC organoids (103), and some promising results have been achieved in animal model experiments (104). In clinical cases, after administering an individualized combination regimen selected based on patient-derived organoid drug sensitivity results, the tumor shrank significantly, and pathological complete cure was achieved through surgical treatment (105). This indicates that organoid models have the potential to assist in personalized treatment and solve the long-standing problem of the difficulty in determining treatment regimens for HCC because of high heterogeneity. Additionally, co-culturing HCC organoids with endothelial cells can reproduce the vascular changes caused by the upregulation of factors such as MCP-1, IL-8, and CXCL16 in the TME (106). This provides an operable *in vitro* model for laboratory research on the TME and drug resistance. However, HCC organoid research still has limitations (107). Firstly, liver cancer organoids mainly consist of tumor cells and lack interactions with stromal cells (such as hepatic stellate cells, vascular endothelial cells) and immune cells, thus failing to truly simulate the tumor immune microenvironment, which limits their application in immunotherapy research (108). Secondly, there is currently no unified assessment standard for “mature” or “functionally normal” organoids, nor a unified culture condition, the construction of organoids lacks standardization and quality control (109); thirdly, the construction success rate of organoids is low and unstable, and they can only maintain activity and function for a limited period of time, and there are challenges in terms of cost and scalability (110). Therefore, although organoid models have therapeutic potential, they currently cannot be widely applied in clinical practice.

### 5.3 Other innovative therapies

Several cutting-edge therapies currently used for other tumors also have therapeutic potential for HCC. Tumor-infiltrating lymphocyte (TIL) therapy has shown high safety for unresectable melanoma and alleviates symptoms even in drug-resistant populations (111). For HCC with a more complex immune environment, the combination of polyclonal TILs with other therapies has therapeutic potential. In prostate cancer and some neuroendocrine tumors of the digestive system, molecular probe-targeted radioligand therapy can significantly prolong overall survival (112). HCC tumors express receptors such as GPC3, ASGPR, and OX40. Consequently, radioligand therapy for HCC may be feasible if highly specific ligands targeting these receptors are identified (113). The TCR-CD3 bispecific immunotherapy for metastatic uveal melanoma also has potential for HCC treatment. After selecting appropriate peptide-HLA targets for HCC, bispecific therapy may also prolong the survival of patients with advanced HCC (114). However, its narrow application range and uncertain toxicity are issues that need to be addressed in subsequent research.

## 6 Conclusion

The evolution of HCC treatment from traditional modalities to immunotherapy-based combinations represents a paradigm shift, yet clinical outcomes remain constrained by the profound heterogeneity of the tumor and the unique tolerogenic nature of the liver microenvironment. Current evidence underscores that overcoming the limitations of first-line immune checkpoint inhibitors requires a deeper disruption of specific resistance mechanisms. Future therapeutic breakthroughs will likely depend on dismantling the “positive feedback loop” of vascular abnormality, hypoxia, and immunosuppression, as well as targeting the physical and chemical barriers created by SPP1+/TREM2+ macrophages and hepatic stellate cells.

Moving forward, the field must transition from a “one-size-fits-all” approach to precision immuno-oncology. The integration of multi-omics with patient-derived organoid (PDO) drug screening offers a viable framework for constructing companion diagnostics that can predict therapeutic response and guide rational drug selection. Furthermore, the clinical translation of emerging modalities—such as TIL therapy, targeted radioligand therapy (RLT), and TCR-CD3 bispecific platforms—holds the potential to address antigen-loss variants and refractory disease. Ultimately, resolving the bottlenecks of primary and acquired resistance through these multi-dimensional strategies will be key to significantly extending survival and improving the quality of life for patients with advanced HCC.
